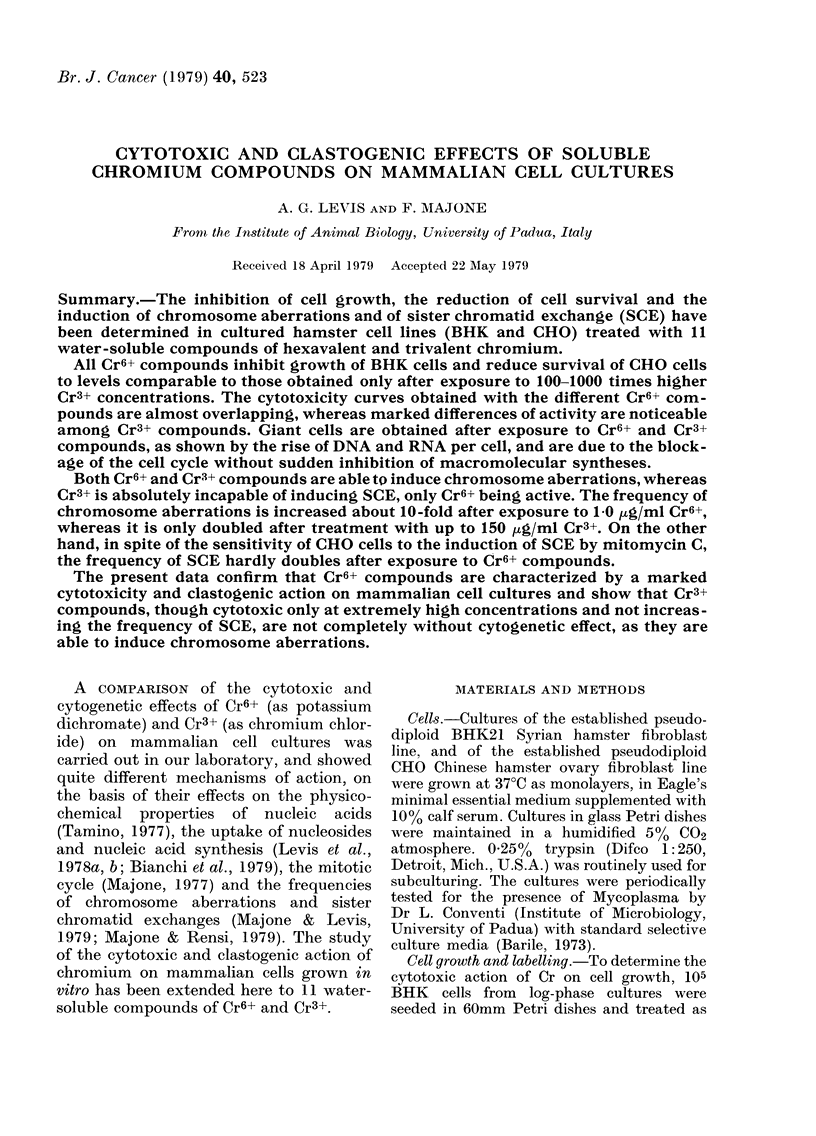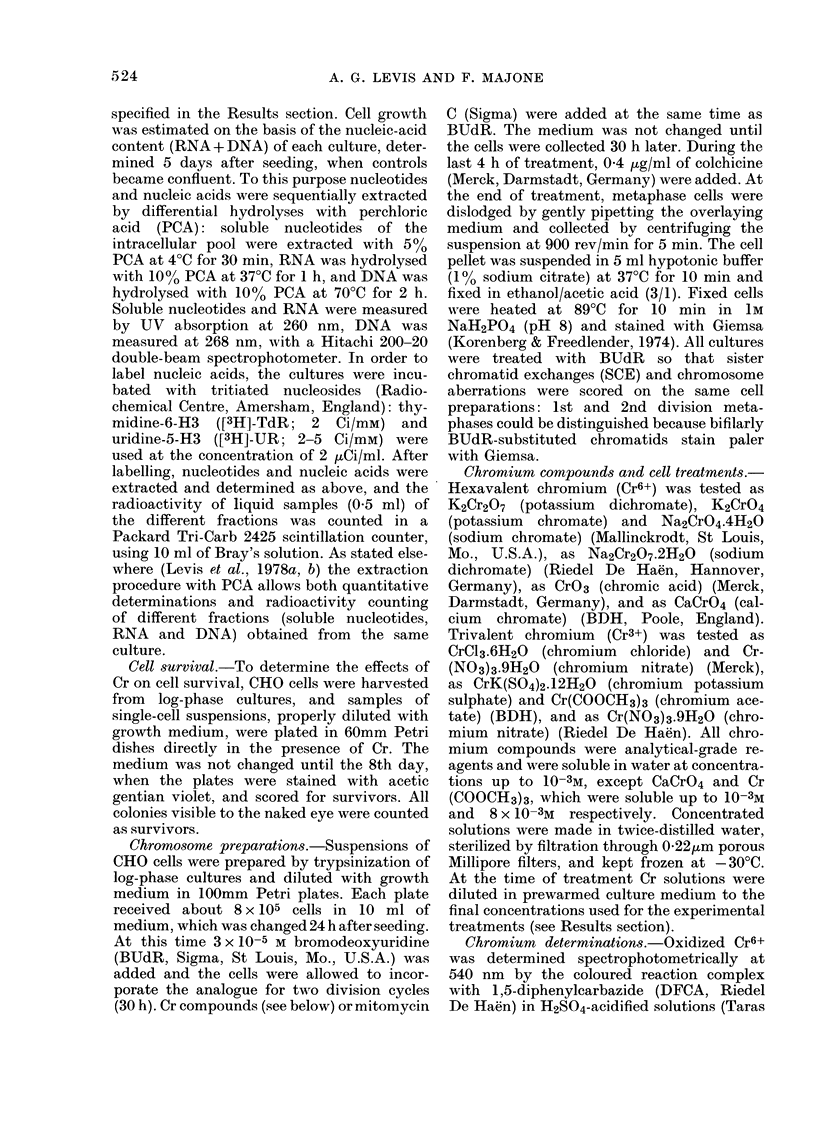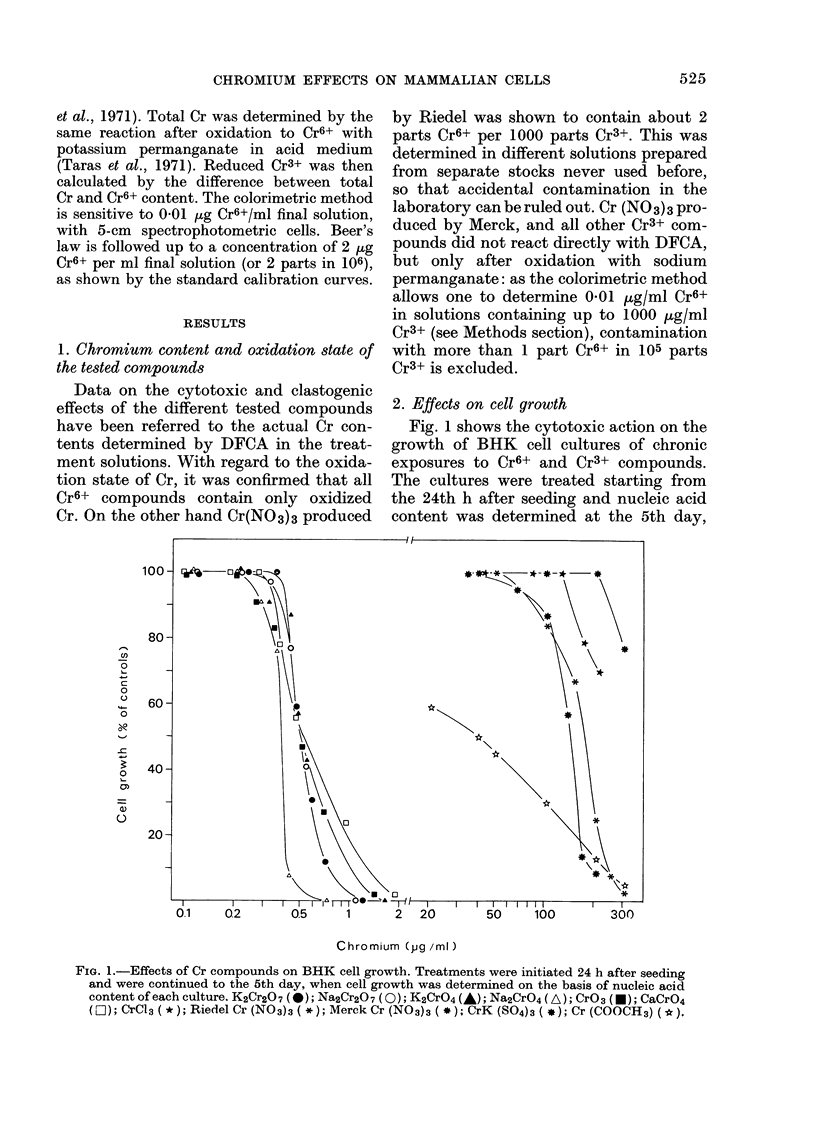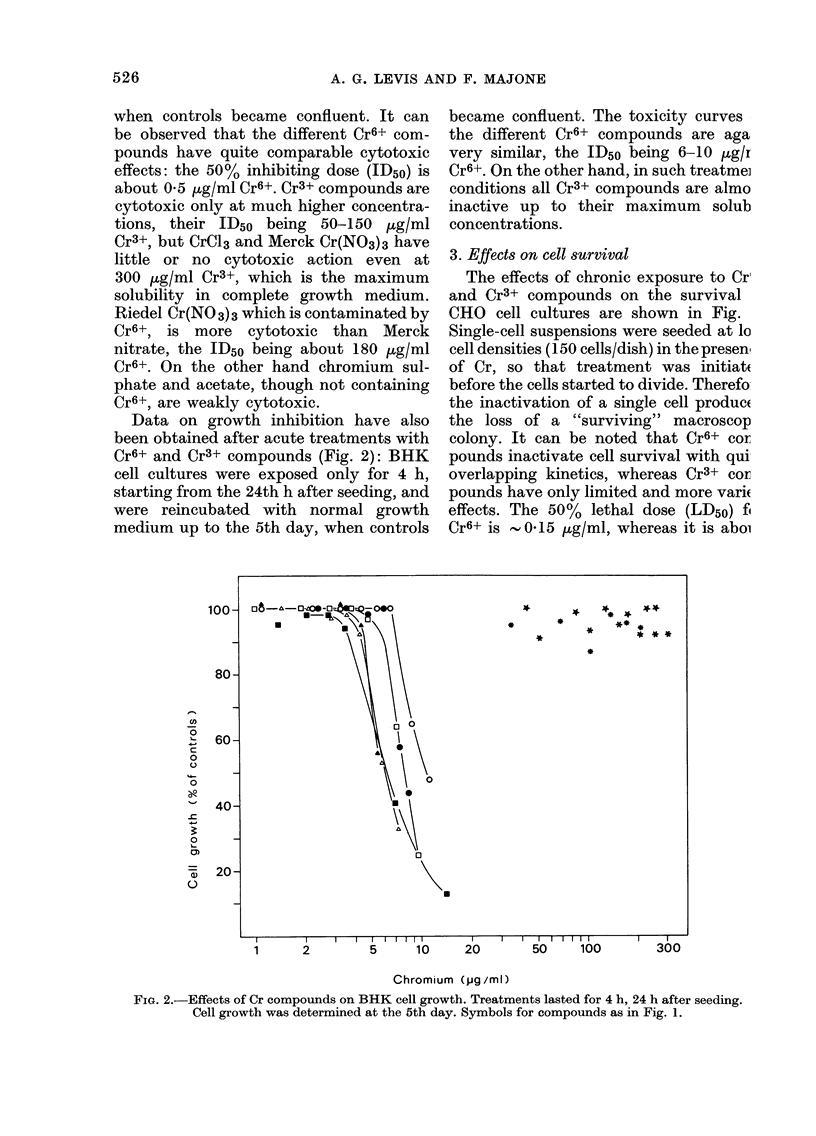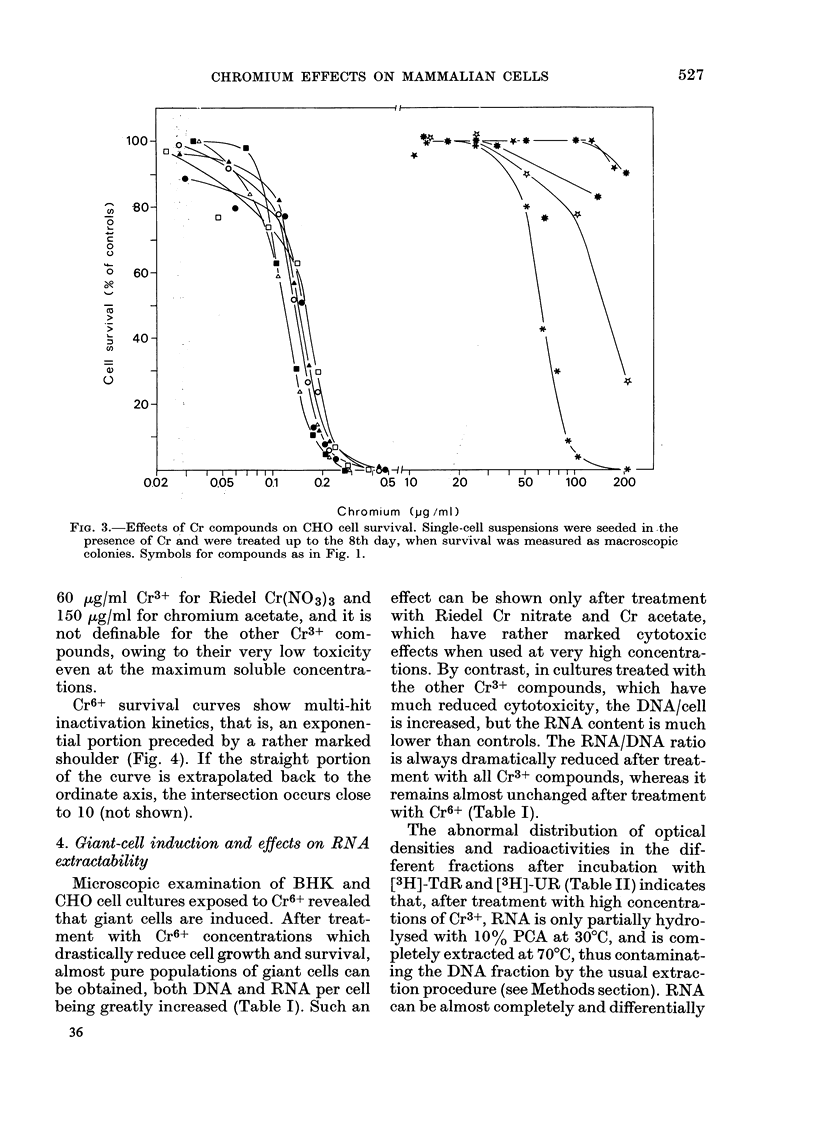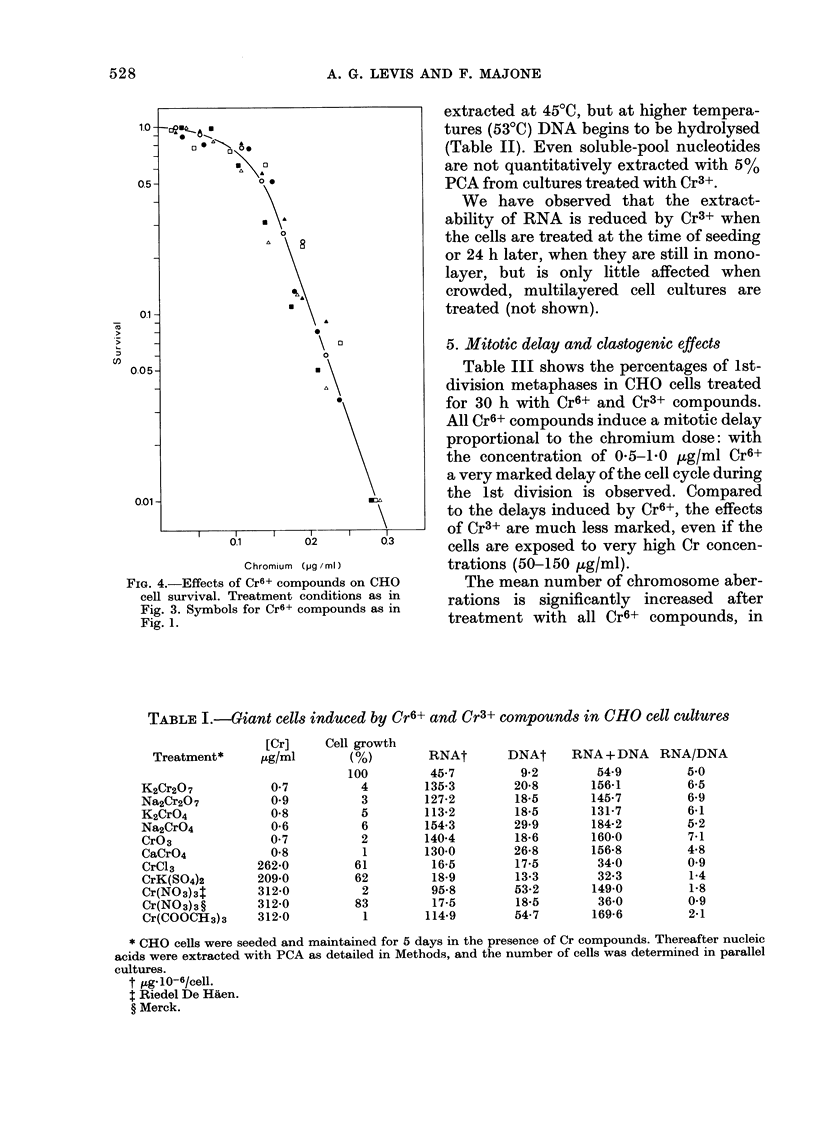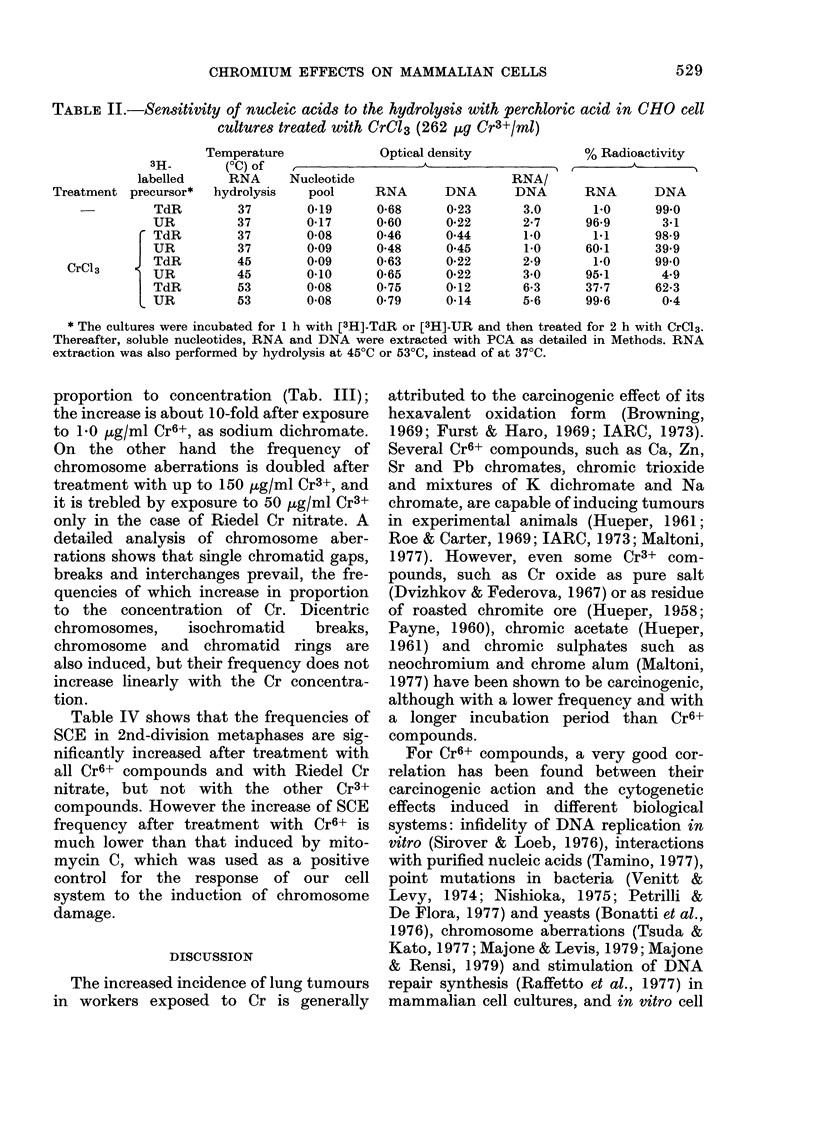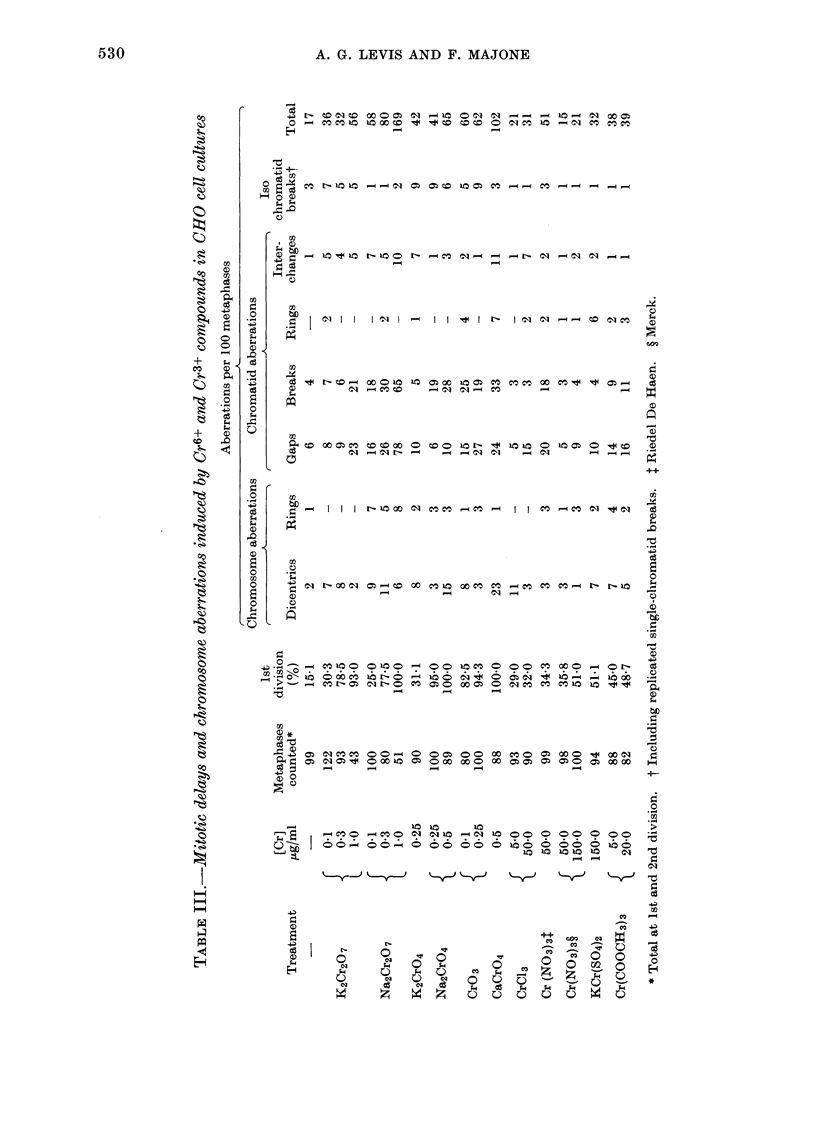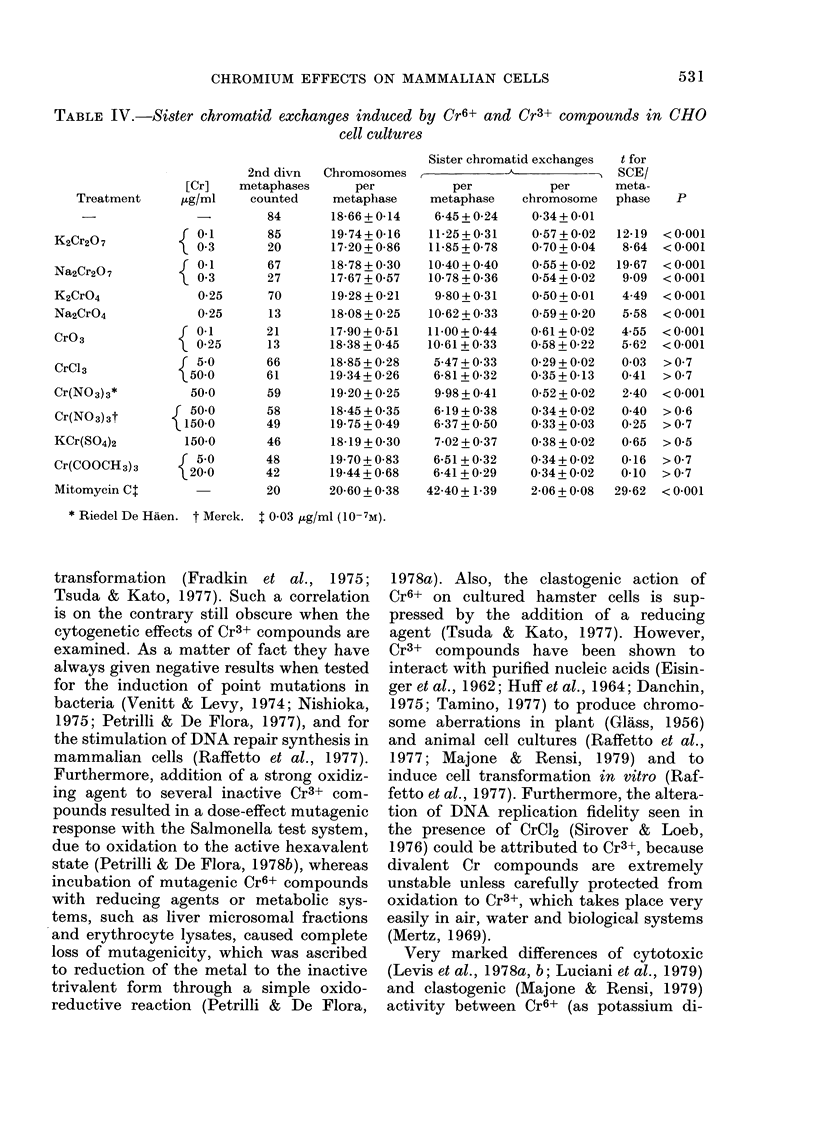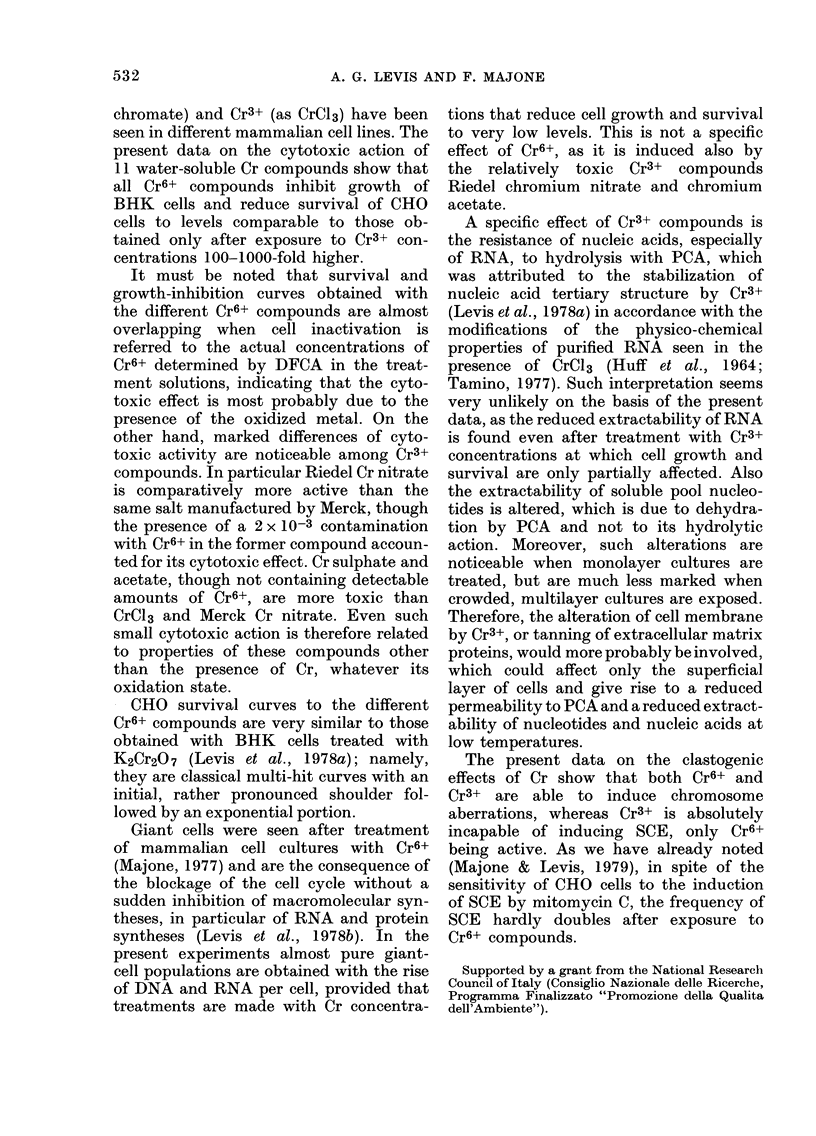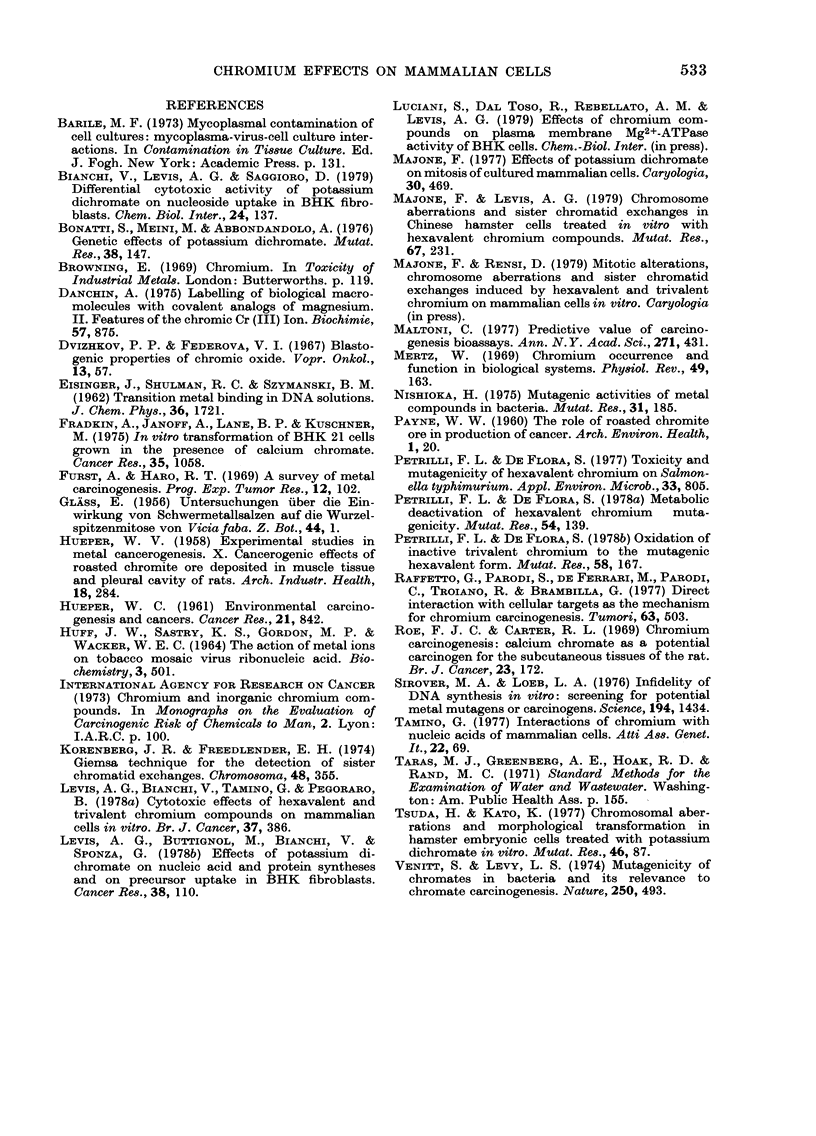# Cytotoxic and clastogenic effects of soluble chromium compounds on mammalian cell cultures.

**DOI:** 10.1038/bjc.1979.217

**Published:** 1979-10

**Authors:** A. G. Levis, F. Majone

## Abstract

The inhibition of cell growth, the reduction of cell survival and the induction of chromosome aberrations and of sister chromatid exchange (SCE) have been determined in cultured hamster cell lines (BHK and CHO) treated with 11 water-soluble compounds of hexavalent and trivalent chromium. All Cr6+ compounds inhibit growth of BHK cells and reduce survival of CHO cells to levels comparable to those obtained only after exposure to 100--1000 times higher Cr3+ concentrations. The cytotoxicity curves obtained with the different Cr6+ compounds are almost overlapping, whereas marked differences of activity are noticeable among Cr3+ compounds. Giant cells are obtained after exposure to Cr6+ and Cr3+ compounds, as shown by the rise of DNA and RNA per cell, and are due to the blockage of the cell cycle without sudden inhibition of macromolecular syntheses. Both Cr6+ and Cr3+ compounds are able to induce chromosome aberrations, whereas Cr3+ is absolutely incapable of inducing SCE, only Cr6+ being active. The frequency of chromosome aberrations is increased about 10-fold after exposure to 1.0 micrograms/ml Cr6+, whereas it is only doubled after treatment with up to 150 micrograms/ml Cr3+. On the other hand, in spite of the sensitivity of CHO cells to the induction of SCE by mitomycin C, the frequency of SCE hardly doubles after exposure to Cr6+ compounds. The present data confirm that Cr6+ compounds are characterized by a marked cytotoxicity and clastogenic action on mammalian cell cultures and show that Cr3+ compounds, though cytotoxic only at extremely high concentrations and not increasing the frequency of SCE, are not completely without cytogenetic effect, as they are able to induce chromosome aberrations.


*Br. J. Cancer* (1979) **40**, 523

### CYTOTOXIC AND CLASTOGENIC EFFECTS OF SOLUBLE CHROMIUM COMPOUNDS ON MAMMALIAN CELL CULTURES

A. G. LEVIS AND F. MAJONE

*From the Institute of Animal Biology, University of Padua, Italy*

Received 18 April 1979 Accepted 22 May 1979

**Summary.—The inhibition of cell growth, the reduction of cell survival and the induction of chromosome aberrations and of sister chromatid exchange (SCE) have been determined in cultured hamster cell lines (BHK and CHO) treated with 11 water-soluble compounds of hexavalent and trivalent chromium.**

**All $Cr^{6+}$ compounds inhibit growth of BHK cells and reduce survival of CHO cells to levels comparable to those obtained only after exposure to 100–1000 times higher $Cr^{3+}$ concentrations. The cytotoxicity curves obtained with the different $Cr^{6+}$ compounds are almost overlapping, whereas marked differences of activity are noticeable among $Cr^{3+}$ compounds. Giant cells are obtained after exposure to $Cr^{6+}$ and $Cr^{3+}$ compounds, as shown by the rise of DNA and RNA per cell, and are due to the blockage of the cell cycle without sudden inhibition of macromolecular syntheses.**

**Both $Cr^{6+}$ and $Cr^{3+}$ compounds are able to induce chromosome aberrations, whereas $Cr^{3+}$ is absolutely incapable of inducing SCE, only $Cr^{6+}$ being active. The frequency of chromosome aberrations is increased about 10-fold after exposure to 1·0 μg/ml $Cr^{6+}$, whereas it is only doubled after treatment with up to 150 μg/ml $Cr^{3+}$. On the other hand, in spite of the sensitivity of CHO cells to the induction of SCE by mitomycin C, the frequency of SCE hardly doubles after exposure to $Cr^{6+}$ compounds.**

**The present data confirm that $Cr^{6+}$ compounds are characterized by a marked cytotoxicity and clastogenic action on mammalian cell cultures and show that $Cr^{3+}$ compounds, though cytotoxic only at extremely high concentrations and not increasing the frequency of SCE, are not completely without cytogenetic effect, as they are able to induce chromosome aberrations.**

A COMPARISON of the cytotoxic and cytogenetic effects of $Cr^{6+}$ (as potassium dichromate) and $Cr^{3+}$ (as chromium chloride) on mammalian cell cultures was carried out in our laboratory, and showed quite different mechanisms of action, on the basis of their effects on the physicochemical properties of nucleic acids (Tamino, 1977), the uptake of nucleosides and nucleic acid synthesis (Levis *et al.*, 1978*a*, *b*; Bianchi *et al.*, 1979), the mitotic cycle (Majone, 1977) and the frequencies of chromosome aberrations and sister chromatid exchanges (Majone & Levis, 1979; Majone & Rensi, 1979). The study of the cytotoxic and clastogenic action of chromium on mammalian cells grown *in vitro* has been extended here to 11 water-soluble compounds of $Cr^{6+}$ and $Cr^{3+}$.

### MATERIALS AND METHODS

*Cells.*—Cultures of the established pseudodiploid BHK21 Syrian hamster fibroblast line, and of the established pseudodiploid CHO Chinese hamster ovary fibroblast line were grown at 37°C as monolayers, in Eagle's minimal essential medium supplemented with 10% calf serum. Cultures in glass Petri dishes were maintained in a humidified 5% $CO_2$ atmosphere. 0·25% trypsin (Difco 1:250, Detroit, Mich., U.S.A.) was routinely used for subculturing. The cultures were periodically tested for the presence of Mycoplasma by Dr L. Conventi (Institute of Microbiology, University of Padua) with standard selective culture media (Barile, 1973).

*Cell growth and labelling.*—To determine the cytotoxic action of Cr on cell growth, $10^5$ BHK cells from log-phase cultures were seeded in 60mm Petri dishes and treated as specified in the Results section. Cell growth was estimated on the basis of the nucleic-acid content (RNA+DNA) of each culture, determined 5 days after seeding, when controls became confluent. To this purpose nucleotides and nucleic acids were sequentially extracted by differential hydrolyses with perchloric acid (PCA): soluble nucleotides of the intracellular pool were extracted with 5% PCA at 4°C for 30 min, RNA was hydrolysed with 10% PCA at 37°C for 1 h, and DNA was hydrolysed with 10% PCA at 70°C for 2 h. Soluble nucleotides and RNA were measured by UV absorption at 260 nm, DNA was measured at 268 nm, with a Hitachi 200–20 double-beam spectrophotometer. In order to label nucleic acids, the cultures were incubated with tritiated nucleosides (Radiochemical Centre, Amersham, England): thymidine-6-H3 ([$^3$H]-TdR; 2 Ci/mM) and uridine-5-H3 ([$^3$H]-UR; 2–5 Ci/mM) were used at the concentration of 2 $\mu$Ci/ml. After labelling, nucleotides and nucleic acids were extracted and determined as above, and the radioactivity of liquid samples (0·5 ml) of the different fractions was counted in a Packard Tri-Carb 2425 scintillation counter, using 10 ml of Bray's solution. As stated elsewhere (Levis *et al.*, 1978*a*, *b*) the extraction procedure with PCA allows both quantitative determinations and radioactivity counting of different fractions (soluble nucleotides, RNA and DNA) obtained from the same culture.

*Cell survival.*—To determine the effects of Cr on cell survival, CHO cells were harvested from log-phase cultures, and samples of single-cell suspensions, properly diluted with growth medium, were plated in 60mm Petri dishes directly in the presence of Cr. The medium was not changed until the 8th day, when the plates were stained with acetic gentian violet, and scored for survivors. All colonies visible to the naked eye were counted as survivors.

*Chromosome preparations.*—Suspensions of CHO cells were prepared by trypsinization of log-phase cultures and diluted with growth medium in 100mm Petri plates. Each plate received about $8\times10^5$ cells in 10 ml of medium, which was changed 24 h after seeding. At this time $3\times10^{-5}$ M bromodeoxyuridine (BUdR, Sigma, St Louis, Mo., U.S.A.) was added and the cells were allowed to incorporate the analogue for two division cycles (30 h). Cr compounds (see below) or mitomycin C (Sigma) were added at the same time as BUdR. The medium was not changed until the cells were collected 30 h later. During the last 4 h of treatment, 0·4 $\mu$g/ml of colchicine (Merck, Darmstadt, Germany) were added. At the end of treatment, metaphase cells were dislodged by gently pipetting the overlaying medium and collected by centrifuging the suspension at 900 rev/min for 5 min. The cell pellet was suspended in 5 ml hypotonic buffer (1% sodium citrate) at 37°C for 10 min and fixed in ethanol/acetic acid (3/1). Fixed cells were heated at 89°C for 10 min in 1M $NaH_2PO_4$ (pH 8) and stained with Giemsa (Korenberg & Freedlender, 1974). All cultures were treated with BUdR so that sister chromatid exchanges (SCE) and chromosome aberrations were scored on the same cell preparations: 1st and 2nd division metaphases could be distinguished because bifilarly BUdR-substituted chromatids stain paler with Giemsa.

*Chromium compounds and cell treatments.*—Hexavalent chromium ($Cr^{6+}$) was tested as $K_2Cr_2O_7$ (potassium dichromate), $K_2CrO_4$ (potassium chromate) and $Na_2CrO_4.4H_2O$ (sodium chromate) (Mallinckrodt, St Louis, Mo., U.S.A.), as $Na_2Cr_2O_7.2H_2O$ (sodium dichromate) (Riedel De Haën, Hannover, Germany), as $CrO_3$ (chromic acid) (Merck, Darmstadt, Germany), and as $CaCrO_4$ (calcium chromate) (BDH, Poole, England). Trivalent chromium ($Cr^{3+}$) was tested as $CrCl_3.6H_2O$ (chromium chloride) and $Cr(NO_3)_3.9H_2O$ (chromium nitrate) (Merck), as $CrK(SO_4)_2.12H_2O$ (chromium potassium sulphate) and $Cr(COOCH_3)_3$ (chromium acetate) (BDH), and as $Cr(NO_3)_3.9H_2O$ (chromium nitrate) (Riedel De Haën). All chromium compounds were analytical-grade reagents and were soluble in water at concentrations up to $10^{-3}$M, except $CaCrO_4$ and $Cr(COOCH_3)_3$, which were soluble up to $10^{-3}$M and $8\times10^{-3}$M respectively. Concentrated solutions were made in twice-distilled water, sterilized by filtration through 0·22$\mu$m porous Millipore filters, and kept frozen at −30°C. At the time of treatment Cr solutions were diluted in prewarmed culture medium to the final concentrations used for the experimental treatments (see Results section).

*Chromium determinations.*—Oxidized $Cr^{6+}$ was determined spectrophotometrically at 540 nm by the coloured reaction complex with 1,5-diphenylcarbazide (DFCA, Riedel De Haën) in $H_2SO_4$-acidified solutions (Taras

*et al.*, 1971). Total Cr was determined by the same reaction after oxidation to $Cr^{6+}$ with potassium permanganate in acid medium (Taras *et al.*, 1971). Reduced $Cr^{3+}$ was then calculated by the difference between total Cr and $Cr^{6+}$ content. The colorimetric method is sensitive to 0·01 μg $Cr^{6+}$/ml final solution, with 5-cm spectrophotometric cells. Beer's law is followed up to a concentration of 2 μg $Cr^{6+}$ per ml final solution (or 2 parts in $10^6$), as shown by the standard calibration curves.

### RESULTS

### 1. *Chromium content and oxidation state of the tested compounds*

Data on the cytotoxic and clastogenic effects of the different tested compounds have been referred to the actual Cr contents determined by DFCA in the treatment solutions. With regard to the oxidation state of Cr, it was confirmed that all $Cr^{6+}$ compounds contain only oxidized Cr. On the other hand $Cr(NO_3)_3$ produced by Riedel was shown to contain about 2 parts $Cr^{6+}$ per 1000 parts $Cr^{3+}$. This was determined in different solutions prepared from separate stocks never used before, so that accidental contamination in the laboratory can be ruled out. Cr $(NO_3)_3$ produced by Merck, and all other $Cr^{3+}$ compounds did not react directly with DFCA, but only after oxidation with sodium permanganate: as the colorimetric method allows one to determine 0·01 μg/ml $Cr^{6+}$ in solutions containing up to 1000 μg/ml $Cr^{3+}$ (see Methods section), contamination with more than 1 part $Cr^{6+}$ in $10^5$ parts $Cr^{3+}$ is excluded.

### 2. *Effects on cell growth*

Fig. 1 shows the cytotoxic action on the growth of BHK cell cultures of chronic exposures to $Cr^{6+}$ and $Cr^{3+}$ compounds. The cultures were treated starting from the 24th h after seeding and nucleic acid content was determined at the 5th day,

FIG. 1.—Effects of Cr compounds on BHK cell growth. Treatments were initiated 24 h after seeding and were continued to the 5th day, when cell growth was determined on the basis of nucleic acid content of each culture. $K_2Cr_2O_7$ (●); $Na_2Cr_2O_7$ (○); $K_2CrO_4$ (▲); $Na_2CrO_4$ (△); $CrO_3$ (■); $CaCrO_4$ (□); $CrCl_3$ (★); Riedel Cr $(NO_3)_3$ (✱); Merck Cr $(NO_3)_3$ (✱); CrK $(SO_4)_3$ (✱); Cr $(COOCH_3)$ (☆).

when controls became confluent. It can be observed that the different $Cr^{6+}$ compounds have quite comparable cytotoxic effects: the 50% inhibiting dose ($ID_{50}$) is about 0·5 μg/ml $Cr^{6+}$. $Cr^{3+}$ compounds are cytotoxic only at much higher concentrations, their $ID_{50}$ being 50–150 μg/ml $Cr^{3+}$, but $CrCl_3$ and Merck $Cr(NO_3)_3$ have little or no cytotoxic action even at 300 μg/ml $Cr^{3+}$, which is the maximum solubility in complete growth medium. Riedel $Cr(NO_3)_3$ which is contaminated by $Cr^{6+}$, is more cytotoxic than Merck nitrate, the $ID_{50}$ being about 180 μg/ml $Cr^{6+}$. On the other hand chromium sulphate and acetate, though not containing $Cr^{6+}$, are weakly cytotoxic.

Data on growth inhibition have also been obtained after acute treatments with $Cr^{6+}$ and $Cr^{3+}$ compounds (Fig. 2): BHK cell cultures were exposed only for 4 h, starting from the 24th h after seeding, and were reincubated with normal growth medium up to the 5th day, when controls became confluent. The toxicity curves the different $Cr^{6+}$ compounds are aga very similar, the $ID_{50}$ being 6–10 μg/ı $Cr^{6+}$. On the other hand, in such treatmeı conditions all $Cr^{3+}$ compounds are almo inactive up to their maximum solub concentrations.

### 3. *Effects on cell survival*

The effects of chronic exposure to Cr' and $Cr^{3+}$ compounds on the survival CHO cell cultures are shown in Fig. Single-cell suspensions were seeded at lo cell densities (150 cells/dish) in the presen of Cr, so that treatment was initiat before the cells started to divide. Therefo the inactivation of a single cell produc the loss of a "surviving" macroscop colony. It can be noted that $Cr^{6+}$ cor pounds inactivate cell survival with qui overlapping kinetics, whereas $Cr^{3+}$ cor pounds have only limited and more vari effects. The 50% lethal dose ($LD_{50}$) f $Cr^{6+}$ is ~0·15 μg/ml, whereas it is abo

FIG. 2.—Effects of Cr compounds on BHK cell growth. Treatments lasted for 4 h, 24 h after seeding. Cell growth was determined at the 5th day. Symbols for compounds as in Fig. 1.

FIG. 3.—Effects of Cr compounds on CHO cell survival. Single-cell suspensions were seeded in the presence of Cr and were treated up to the 8th day, when survival was measured as macroscopic colonies. Symbols for compounds as in Fig. 1.

60 μg/ml $Cr^{3+}$ for Riedel $Cr(NO_3)_3$ and 150 μg/ml for chromium acetate, and it is not definable for the other $Cr^{3+}$ compounds, owing to their very low toxicity even at the maximum soluble concentrations.

$Cr^{6+}$ survival curves show multi-hit inactivation kinetics, that is, an exponential portion preceded by a rather marked shoulder (Fig. 4). If the straight portion of the curve is extrapolated back to the ordinate axis, the intersection occurs close to 10 (not shown).

### 4. *Giant-cell induction and effects on RNA extractability*

Microscopic examination of BHK and CHO cell cultures exposed to $Cr^{6+}$ revealed that giant cells are induced. After treatment with $Cr^{6+}$ concentrations which drastically reduce cell growth and survival, almost pure populations of giant cells can be obtained, both DNA and RNA per cell being greatly increased (Table I). Such an effect can be shown only after treatment with Riedel Cr nitrate and Cr acetate, which have rather marked cytotoxic effects when used at very high concentrations. By contrast, in cultures treated with the other $Cr^{3+}$ compounds, which have much reduced cytotoxicity, the DNA/cell is increased, but the RNA content is much lower than controls. The RNA/DNA ratio is always dramatically reduced after treatment with all $Cr^{3+}$ compounds, whereas it remains almost unchanged after treatment with $Cr^{6+}$ (Table I).

The abnormal distribution of optical densities and radioactivities in the different fractions after incubation with [$^3$H]-TdR and [$^3$H]-UR (Table II) indicates that, after treatment with high concentrations of $Cr^{3+}$, RNA is only partially hydrolysed with 10% PCA at 30°C, and is completely extracted at 70°C, thus contaminating the DNA fraction by the usual extraction procedure (see Methods section). RNA can be almost completely and differentially

FIG. 4.—Effects of $Cr^{6+}$ compounds on CHO cell survival. Treatment conditions as in Fig. 3. Symbols for $Cr^{6+}$ compounds as in Fig. 1.

extracted at 45°C, but at higher temperatures (53°C) DNA begins to be hydrolysed (Table II). Even soluble-pool nucleotides are not quantitatively extracted with 5% PCA from cultures treated with $Cr^{3+}$.

We have observed that the extractability of RNA is reduced by $Cr^{3+}$ when the cells are treated at the time of seeding or 24 h later, when they are still in monolayer, but is only little affected when crowded, multilayered cell cultures are treated (not shown).

### 5. *Mitotic delay and clastogenic effects*

Table III shows the percentages of 1st-division metaphases in CHO cells treated for 30 h with $Cr^{6+}$ and $Cr^{3+}$ compounds. All $Cr^{6+}$ compounds induce a mitotic delay proportional to the chromium dose: with the concentration of 0·5–1·0 μg/ml $Cr^{6+}$ a very marked delay of the cell cycle during the 1st division is observed. Compared to the delays induced by $Cr^{6+}$, the effects of $Cr^{3+}$ are much less marked, even if the cells are exposed to very high Cr concentrations (50–150 μg/ml).

The mean number of chromosome aberrations is significantly increased after treatment with all $Cr^{6+}$ compounds, in

TABLE I.—*Giant cells induced by $Cr^{6+}$ and $Cr^{3+}$ compounds in CHO cell cultures*

| Treatment* | [Cr] μg/ml | Cell growth (%) | RNA† | DNA† | RNA+DNA | RNA/DNA |
|---|---|---|---|---|---|---|
| | | 100 | 45·7 | 9·2 | 54·9 | 5·0 |
| $K_2Cr_2O_7$ | 0·7 | 4 | 135·3 | 20·8 | 156·1 | 6·5 |
| $Na_2Cr_2O_7$ | 0·9 | 3 | 127·2 | 18·5 | 145·7 | 6·9 |
| $K_2CrO_4$ | 0·8 | 5 | 113·2 | 18·5 | 131·7 | 6·1 |
| $Na_2CrO_4$ | 0·6 | 6 | 154·3 | 29·9 | 184·2 | 5·2 |
| $CrO_3$ | 0·7 | 2 | 140·4 | 18·6 | 160·0 | 7·1 |
| $CaCrO_4$ | 0·8 | 1 | 130·0 | 26·8 | 156·8 | 4·8 |
| $CrCl_3$ | 262·0 | 61 | 16·5 | 17·5 | 34·0 | 0·9 |
| $CrK(SO_4)_2$ | 209·0 | 62 | 18·9 | 13·3 | 32·3 | 1·4 |
| $Cr(NO_3)_3$‡ | 312·0 | 2 | 95·8 | 53·2 | 149·0 | 1·8 |
| $Cr(NO_3)_3$§ | 312·0 | 83 | 17·5 | 18·5 | 36·0 | 0·9 |
| $Cr(COOCH_3)_3$ | 312·0 | 1 | 114·9 | 54·7 | 169·6 | 2·1 |

* CHO cells were seeded and maintained for 5 days in the presence of Cr compounds. Thereafter nucleic acids were extracted with PCA as detailed in Methods, and the number of cells was determined in parallel cultures.

† $\mu g \cdot 10^{-6}$/cell.

‡ Riedel De Häen.

§ Merck.

TABLE II.—*Sensitivity of nucleic acids to the hydrolysis with perchloric acid in CHO cell cultures treated with $CrCl_3$ (262 μg $Cr^{3+}$/ml)*

| Treatment | $^3$H-labelled precursor* | Temperature (°C) of RNA hydrolysis | Optical density: Nucleotide pool | Optical density: RNA | Optical density: DNA | Optical density: RNA/DNA | % Radioactivity: RNA | % Radioactivity: DNA |
|---|---|---|---|---|---|---|---|---|
| — | TdR | 37 | 0·19 | 0·68 | 0·23 | 3.0 | 1·0 | 99·0 |
| | UR | 37 | 0·17 | 0·60 | 0·22 | 2·7 | 96·9 | 3·1 |
| $CrCl_3$ | TdR | 37 | 0·08 | 0·46 | 0·44 | 1·0 | 1·1 | 98·9 |
| | UR | 37 | 0·09 | 0·48 | 0·45 | 1·0 | 60·1 | 39·9 |
| | TdR | 45 | 0·09 | 0·63 | 0·22 | 2·9 | 1·0 | 99·0 |
| | UR | 45 | 0·10 | 0·65 | 0·22 | 3·0 | 95·1 | 4·9 |
| | TdR | 53 | 0·08 | 0·75 | 0·12 | 6·3 | 37·7 | 62·3 |
| | UR | 53 | 0·08 | 0·79 | 0·14 | 5·6 | 99·6 | 0·4 |

* The cultures were incubated for 1 h with [$^3$H]-TdR or [$^3$H]-UR and then treated for 2 h with $CrCl_3$. Thereafter, soluble nucleotides, RNA and DNA were extracted with PCA as detailed in Methods. RNA extraction was also performed by hydrolysis at 45°C or 53°C, instead of at 37°C.

proportion to concentration (Tab. III); the increase is about 10-fold after exposure to 1·0 μg/ml $Cr^{6+}$, as sodium dichromate. On the other hand the frequency of chromosome aberrations is doubled after treatment with up to 150 μg/ml $Cr^{3+}$, and it is trebled by exposure to 50 μg/ml $Cr^{3+}$ only in the case of Riedel Cr nitrate. A detailed analysis of chromosome aberrations shows that single chromatid gaps, breaks and interchanges prevail, the frequencies of which increase in proportion to the concentration of Cr. Dicentric chromosomes, isochromatid breaks, chromosome and chromatid rings are also induced, but their frequency does not increase linearly with the Cr concentration.

Table IV shows that the frequencies of SCE in 2nd-division metaphases are significantly increased after treatment with all $Cr^{6+}$ compounds and with Riedel Cr nitrate, but not with the other $Cr^{3+}$ compounds. However the increase of SCE frequency after treatment with $Cr^{6+}$ is much lower than that induced by mitomycin C, which was used as a positive control for the response of our cell system to the induction of chromosome damage.

### DISCUSSION

The increased incidence of lung tumours in workers exposed to Cr is generally attributed to the carcinogenic effect of its hexavalent oxidation form (Browning, 1969; Furst & Haro, 1969; IARC, 1973). Several $Cr^{6+}$ compounds, such as Ca, Zn, Sr and Pb chromates, chromic trioxide and mixtures of K dichromate and Na chromate, are capable of inducing tumours in experimental animals (Hueper, 1961; Roe & Carter, 1969; IARC, 1973; Maltoni, 1977). However, even some $Cr^{3+}$ compounds, such as Cr oxide as pure salt (Dvizhkov & Federova, 1967) or as residue of roasted chromite ore (Hueper, 1958; Payne, 1960), chromic acetate (Hueper, 1961) and chromic sulphates such as neochromium and chrome alum (Maltoni, 1977) have been shown to be carcinogenic, although with a lower frequency and with a longer incubation period than $Cr^{6+}$ compounds.

For $Cr^{6+}$ compounds, a very good correlation has been found between their carcinogenic action and the cytogenetic effects induced in different biological systems: infidelity of DNA replication *in vitro* (Sirover & Loeb, 1976), interactions with purified nucleic acids (Tamino, 1977), point mutations in bacteria (Venitt & Levy, 1974; Nishioka, 1975; Petrilli & De Flora, 1977) and yeasts (Bonatti *et al.*, 1976), chromosome aberrations (Tsuda & Kato, 1977; Majone & Levis, 1979; Majone & Rensi, 1979) and stimulation of DNA repair synthesis (Raffetto *et al.*, 1977) in mammalian cell cultures, and *in vitro* cell

TABLE III.—*Mitotic delays and chromosome aberrations induced by $Cr^{6+}$ and $Cr^{3+}$ compounds in CHO cell cultures*

| Treatment | [Cr] μg/ml | Metaphases counted* | 1st division (%) | Aberrations per 100 metaphases: Chromosome aberrations | | Chromatid aberrations | | | | Iso chromatid breaks† | Total |
|---|---|---|---|---|---|---|---|---|---|---|---|
| | | | | Dicentrics | Rings | Gaps | Breaks | Rings | Inter-changes | | |
| — | — | 99 | 15·1 | 2 | 1 | 6 | 4 | — | 1 | 3 | 17 |
| $K_2Cr_2O_7$ | 0·1 | 122 | 30·3 | 7 | – | 8 | 7 | 2 | 5 | 7 | 36 |
| | 0·3 | 93 | 78·5 | 8 | – | 9 | 6 | – | 4 | 5 | 32 |
| | 1·0 | 43 | 93·0 | 2 | – | 23 | 21 | – | 5 | 5 | 56 |
| $Na_2Cr_2O_7$ | 0·1 | 100 | 25·0 | 9 | 7 | 16 | 18 | – | 7 | 1 | 58 |
| | 0·3 | 80 | 77·5 | 11 | 5 | 26 | 30 | 2 | 5 | 1 | 80 |
| | 1·0 | 51 | 100·0 | 6 | 8 | 78 | 65 | – | 10 | 2 | 169 |
| $K_2CrO_4$ | 0·25 | 90 | 31·1 | 8 | 2 | 10 | 5 | 1 | 7 | 9 | 42 |
| $Na_2CrO_4$ | 0·25 | 100 | 95·0 | 3 | 3 | 6 | 19 | – | 1 | 9 | 41 |
| | 0·5 | 89 | 100·0 | 15 | 3 | 10 | 28 | – | 3 | 6 | 65 |
| $CrO_3$ | 0·1 | 80 | 82·5 | 8 | 1 | 15 | 25 | 4 | 2 | 5 | 60 |
| | 0·25 | 100 | 94·3 | 3 | 3 | 27 | 19 | – | 1 | 9 | 62 |
| $CaCrO_4$ | 0·5 | 88 | 100·0 | 23 | 1 | 24 | 33 | 7 | 11 | 3 | 102 |
| $CrCl_3$ | 5·0 | 93 | 29·0 | 11 | – | 5 | 3 | – | 1 | 1 | 21 |
| | 50·0 | 90 | 32·0 | 3 | – | 15 | 3 | 2 | 7 | 1 | 31 |
| $Cr(NO_3)_3$‡ | 50·0 | 99 | 34·3 | 3 | 3 | 20 | 18 | 2 | 2 | 3 | 51 |
| $Cr(NO_3)_3$§ | 50·0 | 98 | 35·8 | 3 | 1 | 5 | 3 | 1 | 1 | 1 | 15 |
| | 150·0 | 100 | 51·0 | 1 | 3 | 9 | 4 | 1 | 2 | 1 | 21 |
| $KCr(SO_4)_2$ | 150·0 | 94 | 51·1 | 7 | 2 | 10 | 4 | 6 | 2 | 1 | 32 |
| $Cr(COOCH_3)_3$ | 5·0 | 88 | 45·0 | 7 | 4 | 14 | 9 | 2 | 1 | 1 | 38 |
| | 20·0 | 82 | 48·7 | 5 | 2 | 16 | 11 | 3 | 1 | 1 | 39 |

* Total at 1st and 2nd division. † Including replicated single-chromatid breaks. ‡ Riedel De Haen. § Merck.

TABLE IV.—*Sister chromatid exchanges induced by $Cr^{6+}$ and $Cr^{3+}$ compounds in CHO cell cultures*

| Treatment | [Cr] μg/ml | 2nd divn metaphases counted | Chromosomes per metaphase | Sister chromatid exchanges per metaphase | Sister chromatid exchanges per chromosome | t for SCE/ metaphase | P |
|---|---|---|---|---|---|---|---|
| — | — | 84 | 18·66 ± 0·14 | 6·45 ± 0·24 | 0·34 ± 0·01 | | |
| $K_2Cr_2O_7$ | 0·1 | 85 | 19·74 ± 0·16 | 11·25 ± 0·31 | 0·57 ± 0·02 | 12·19 | < 0·001 |
| | 0·3 | 20 | 17·20 ± 0·86 | 11·85 ± 0·78 | 0·70 ± 0·04 | 8·64 | < 0·001 |
| $Na_2Cr_2O_7$ | 0·1 | 67 | 18·78 ± 0·30 | 10·40 ± 0·40 | 0·55 ± 0·02 | 19·67 | < 0·001 |
| | 0·3 | 27 | 17·67 ± 0·57 | 10·78 ± 0·36 | 0·54 ± 0·02 | 9·09 | < 0·001 |
| $K_2CrO_4$ | 0·25 | 70 | 19·28 ± 0·21 | 9·80 ± 0·31 | 0·50 ± 0·01 | 4·49 | < 0·001 |
| $Na_2CrO_4$ | 0·25 | 13 | 18·08 ± 0·25 | 10·62 ± 0·33 | 0·59 ± 0·20 | 5·58 | < 0·001 |
| $CrO_3$ | 0·1 | 21 | 17·90 ± 0·51 | 11·00 ± 0·44 | 0·61 ± 0·02 | 4·55 | < 0·001 |
| | 0·25 | 13 | 18·38 ± 0·45 | 10·61 ± 0·33 | 0·58 ± 0·22 | 5·62 | < 0·001 |
| $CrCl_3$ | 5·0 | 66 | 18·85 ± 0·28 | 5·47 ± 0·33 | 0·29 ± 0·02 | 0·03 | > 0·7 |
| | 50·0 | 61 | 19·34 ± 0·26 | 6·81 ± 0·32 | 0·35 ± 0·13 | 0·41 | > 0·7 |
| $Cr(NO_3)_3$* | 50·0 | 59 | 19·20 ± 0·25 | 9·98 ± 0·41 | 0·52 ± 0·02 | 2·40 | < 0·001 |
| $Cr(NO_3)_3$† | 50·0 | 58 | 18·45 ± 0·35 | 6·19 ± 0·38 | 0·34 ± 0·02 | 0·40 | > 0·6 |
| | 150·0 | 49 | 19·75 ± 0·49 | 6·37 ± 0·50 | 0·33 ± 0·03 | 0·25 | > 0·7 |
| $KCr(SO_4)_2$ | 150·0 | 46 | 18·19 ± 0·30 | 7·02 ± 0·37 | 0·38 ± 0·02 | 0·65 | > 0·5 |
| $Cr(COOCH_3)_3$ | 5·0 | 48 | 19·70 ± 0·83 | 6·51 ± 0·32 | 0·34 ± 0·02 | 0·16 | > 0·7 |
| | 20·0 | 42 | 19·44 ± 0·68 | 6·41 ± 0·29 | 0·34 ± 0·02 | 0·10 | > 0·7 |
| Mitomycin C‡ | — | 20 | 20·60 ± 0·38 | 42·40 ± 1·39 | 2·06 ± 0·08 | 29·62 | < 0·001 |

* Riedel De Häen. † Merck. ‡ 0·03 μg/ml ($10^{-7}$M).

transformation (Fradkin *et al.*, 1975; Tsuda & Kato, 1977). Such a correlation is on the contrary still obscure when the cytogenetic effects of $Cr^{3+}$ compounds are examined. As a matter of fact they have always given negative results when tested for the induction of point mutations in bacteria (Venitt & Levy, 1974; Nishioka, 1975; Petrilli & De Flora, 1977), and for the stimulation of DNA repair synthesis in mammalian cells (Raffetto *et al.*, 1977). Furthermore, addition of a strong oxidizing agent to several inactive $Cr^{3+}$ compounds resulted in a dose-effect mutagenic response with the Salmonella test system, due to oxidation to the active hexavalent state (Petrilli & De Flora, 1978*b*), whereas incubation of mutagenic $Cr^{6+}$ compounds with reducing agents or metabolic systems, such as liver microsomal fractions and erythrocyte lysates, caused complete loss of mutagenicity, which was ascribed to reduction of the metal to the inactive trivalent form through a simple oxido-reductive reaction (Petrilli & De Flora, 1978*a*). Also, the clastogenic action of $Cr^{6+}$ on cultured hamster cells is suppressed by the addition of a reducing agent (Tsuda & Kato, 1977). However, $Cr^{3+}$ compounds have been shown to interact with purified nucleic acids (Eisinger *et al.*, 1962; Huff *et al.*, 1964; Danchin, 1975; Tamino, 1977) to produce chromosome aberrations in plant (Gläss, 1956) and animal cell cultures (Raffetto *et al.*, 1977; Majone & Rensi, 1979) and to induce cell transformation *in vitro* (Raffetto *et al.*, 1977). Furthermore, the alteration of DNA replication fidelity seen in the presence of $CrCl_2$ (Sirover & Loeb, 1976) could be attributed to $Cr^{3+}$, because divalent Cr compounds are extremely unstable unless carefully protected from oxidation to $Cr^{3+}$, which takes place very easily in air, water and biological systems (Mertz, 1969).

Very marked differences of cytotoxic (Levis *et al.*, 1978*a*, *b*; Luciani *et al.*, 1979) and clastogenic (Majone & Rensi, 1979) activity between $Cr^{6+}$ (as potassium dichromate) and $Cr^{3+}$ (as $CrCl_3$) have been seen in different mammalian cell lines. The present data on the cytotoxic action of 11 water-soluble Cr compounds show that all $Cr^{6+}$ compounds inhibit growth of BHK cells and reduce survival of CHO cells to levels comparable to those obtained only after exposure to $Cr^{3+}$ concentrations 100–1000-fold higher.

It must be noted that survival and growth-inhibition curves obtained with the different $Cr^{6+}$ compounds are almost overlapping when cell inactivation is referred to the actual concentrations of $Cr^{6+}$ determined by DFCA in the treatment solutions, indicating that the cytotoxic effect is most probably due to the presence of the oxidized metal. On the other hand, marked differences of cytotoxic activity are noticeable among $Cr^{3+}$ compounds. In particular Riedel Cr nitrate is comparatively more active than the same salt manufactured by Merck, though the presence of a $2 \times 10^{-3}$ contamination with $Cr^{6+}$ in the former compound accounted for its cytotoxic effect. Cr sulphate and acetate, though not containing detectable amounts of $Cr^{6+}$, are more toxic than $CrCl_3$ and Merck Cr nitrate. Even such small cytotoxic action is therefore related to properties of these compounds other than the presence of Cr, whatever its oxidation state.

CHO survival curves to the different $Cr^{6+}$ compounds are very similar to those obtained with BHK cells treated with $K_2Cr_2O_7$ (Levis *et al.*, 1978*a*); namely, they are classical multi-hit curves with an initial, rather pronounced shoulder followed by an exponential portion.

Giant cells were seen after treatment of mammalian cell cultures with $Cr^{6+}$ (Majone, 1977) and are the consequence of the blockage of the cell cycle without a sudden inhibition of macromolecular syntheses, in particular of RNA and protein syntheses (Levis *et al.*, 1978*b*). In the present experiments almost pure giant-cell populations are obtained with the rise of DNA and RNA per cell, provided that treatments are made with Cr concentrations that reduce cell growth and survival to very low levels. This is not a specific effect of $Cr^{6+}$, as it is induced also by the relatively toxic $Cr^{3+}$ compounds Riedel chromium nitrate and chromium acetate.

A specific effect of $Cr^{3+}$ compounds is the resistance of nucleic acids, especially of RNA, to hydrolysis with PCA, which was attributed to the stabilization of nucleic acid tertiary structure by $Cr^{3+}$ (Levis *et al.*, 1978*a*) in accordance with the modifications of the physico-chemical properties of purified RNA seen in the presence of $CrCl_3$ (Huff *et al.*, 1964; Tamino, 1977). Such interpretation seems very unlikely on the basis of the present data, as the reduced extractability of RNA is found even after treatment with $Cr^{3+}$ concentrations at which cell growth and survival are only partially affected. Also the extractability of soluble pool nucleotides is altered, which is due to dehydration by PCA and not to its hydrolytic action. Moreover, such alterations are noticeable when monolayer cultures are treated, but are much less marked when crowded, multilayer cultures are exposed. Therefore, the alteration of cell membrane by $Cr^{3+}$, or tanning of extracellular matrix proteins, would more probably be involved, which could affect only the superficial layer of cells and give rise to a reduced permeability to PCA and a reduced extractability of nucleotides and nucleic acids at low temperatures.

The present data on the clastogenic effects of Cr show that both $Cr^{6+}$ and $Cr^{3+}$ are able to induce chromosome aberrations, whereas $Cr^{3+}$ is absolutely incapable of inducing SCE, only $Cr^{6+}$ being active. As we have already noted (Majone & Levis, 1979), in spite of the sensitivity of CHO cells to the induction of SCE by mitomycin C, the frequency of SCE hardly doubles after exposure to $Cr^{6+}$ compounds.

Supported by a grant from the National Research Council of Italy (Consiglio Nazionale delle Ricerche, Programma Finalizzato "Promozione della Qualita dell'Ambiente").

### REFERENCES

Barile, M. F. (1973) Mycoplasmal contamination of cell cultures: mycoplasma-virus-cell culture interactions. In *Contamination in Tissue Culture*. Ed. J. Fogh. New York: Academic Press. p. 131.

Bianchi, V., Levis, A. G. & Saggioro, D. (1979) Differential cytotoxic activity of potassium dichromate on nucleoside uptake in BHK fibroblasts. *Chem. Biol. Inter.*, **24**, 137.

Bonatti, S., Meini, M. & Abbondandolo, A. (1976) Genetic effects of potassium dichromate. *Mutat. Res.*, **38**, 147.

Browning, E. (1969) Chromium. In *Toxicity of Industrial Metals*. London: Butterworths. p. 119.

Danchin, A. (1975) Labelling of biological macromolecules with covalent analogs of magnesium. II. Features of the chromic Cr (III) Ion. *Biochimie*, **57**, 875.

Dvizhkov, P. P. & Federova, V. I. (1967) Blastogenic properties of chromic oxide. *Vopr. Onkol.*, **13**, 57.

Eisinger, J., Shulman, R. C. & Szymanski, B. M. (1962) Transition metal binding in DNA solutions. *J. Chem. Phys.*, **36**, 1721.

Fradkin, A., Janoff, A., Lane, B. P. & Kuschner, M. (1975) *In vitro* transformation of BHK 21 cells grown in the presence of calcium chromate. *Cancer Res.*, **35**, 1058.

Furst, A. & Haro, R. T. (1969) A survey of metal carcinogenesis. *Prog. Exp. Tumor Res.*, **12**, 102.

Gläss, E. (1956) Untersuchungen über die Einwirkung von Schwermetallsalzen auf die Wurzelspitzenmitose von *Vicia faba. Z. Bot.*, **44**, 1.

Hueper, W. V. (1958) Experimental studies in metal cancerogenesis. X. Cancerogenic effects of roasted chromite ore deposited in muscle tissue and pleural cavity of rats. *Arch. Industr. Health*, **18**, 284.

Hueper, W. C. (1961) Environmental carcinogenesis and cancers. *Cancer Res.*, **21**, 842.

Huff, J. W., Sastry, K. S., Gordon, M. P. & Wacker, W. E. C. (1964) The action of metal ions on tobacco mosaic virus ribonucleic acid. *Biochemistry*, **3**, 501.

International Agency for Research on Cancer (1973) Chromium and inorganic chromium compounds. In *Monographs on the Evaluation of Carcinogenic Risk of Chemicals to Man*, **2**. Lyon: I.A.R.C. p. 100.

Korenberg, J. R. & Freedlender, E. H. (1974) Giemsa technique for the detection of sister chromatid exchanges. *Chromosoma*, **48**, 355.

Levis, A. G., Bianchi, V., Tamino, G. & Pegoraro, B. (1978*a*) Cytotoxic effects of hexavalent and trivalent chromium compounds on mammalian cells *in vitro*. *Br. J. Cancer*, **37**, 386.

Levis, A. G., Buttignol, M., Bianchi, V. & Sponza, G. (1978*b*) Effects of potassium dichromate on nucleic acid and protein syntheses and on precursor uptake in BHK fibroblasts. *Cancer Res.*, **38**, 110.

Luciani, S., Dal Toso, R., Rebellato, A. M. & Levis, A. G. (1979) Effects of chromium compounds on plasma membrane $Mg^{2+}$-ATPase activity of BHK cells. *Chem.-Biol. Inter.* (in press).

Majone, F. (1977) Effects of potassium dichromate on mitosis of cultured mammalian cells. *Caryologia*, **30**, 469.

Majone, F. & Levis, A. G. (1979) Chromosome aberrations and sister chromatid exchanges in Chinese hamster cells treated *in vitro* with hexavalent chromium compounds. *Mutat. Res.*, **67**, 231.

Majone, F. & Rensi, D. (1979) Mitotic alterations, chromosome aberrations and sister chromatid exchanges induced by hexavalent and trivalent chromium on mammalian cells *in vitro*. *Caryologia* (in press).

Maltoni, C. (1977) Predictive value of carcinogenesis bioassays. *Ann. N.Y. Acad. Sci.*, **271**, 431.

Mertz, W. (1969) Chromium occurrence and function in biological systems. *Physiol. Rev.*, **49**, 163.

Nishioka, H. (1975) Mutagenic activities of metal compounds in bacteria. *Mutat. Res.*, **31**, 185.

Payne, W. W. (1960) The role of roasted chromite ore in production of cancer. *Arch. Environ. Health*, **1**, 20.

Petrilli, F. L. & De Flora, S. (1977) Toxicity and mutagenicity of hexavalent chromium on *Salmonella typhimurium*. *Appl. Environ. Microb.*, **33**, 805.

Petrilli, F. L. & De Flora, S. (1978*a*) Metabolic deactivation of hexavalent chromium mutagenicity. *Mutat. Res.*, **54**, 139.

Petrilli, F. L. & De Flora, S. (1978*b*) Oxidation of inactive trivalent chromium to the mutagenic hexavalent form. *Mutat. Res.*, **58**, 167.

Raffetto, G., Parodi, S., de Ferrari, M., Parodi, C., Troiano, R. & Brambilla, G. (1977) Direct interaction with cellular targets as the mechanism for chromium carcinogenesis. *Tumori*, **63**, 503.

Roe, F. J. C. & Carter, R. L. (1969) Chromium carcinogenesis: calcium chromate as a potential carcinogen for the subcutaneous tissues of the rat. *Br. J. Cancer*, **23**, 172.

Sirover, M. A. & Loeb, L. A. (1976) Infidelity of DNA synthesis *in vitro*: screening for potential metal mutagens or carcinogens. *Science*, **194**, 1434.

Tamino, G. (1977) Interactions of chromium with nucleic acids of mammalian cells. *Atti Ass. Genet. It.*, **22**, 69.

Taras, M. J., Greenberg, A. E., Hoak, R. D. & Rand, M. C. (1971) *Standard Methods for the Examination of Water and Wastewater*. Washington: Am. Public Health Ass. p. 155.

Tsuda, H. & Kato, K. (1977) Chromosomal aberrations and morphological transformation in hamster embryonic cells treated with potassium dichromate *in vitro*. *Mutat. Res.*, **46**, 87.

Venitt, S. & Levy, L. S. (1974) Mutagenicity of chromates in bacteria and its relevance to chromate carcinogenesis. *Nature*, **250**, 493.